# Reliability and Factor Structure of the Well-Being and Respect for Human Rights Questionnaire in Measuring Caregivers' Perception

**DOI:** 10.2174/0117450179310030240703061409

**Published:** 2024-07-10

**Authors:** Michela Atzeni, Mauro G. Carta, Diego Primavera, Cesar Ivan Aviles Gonzales, Maura Galletta, Sonia Marchegiani, Giorgio Carboni, Federica Sancassiani, Marcello Nonnis, Antonio Urban, Elisa Cantone, Antonio E. Nardi, Antonio Preti

**Affiliations:** 1 International Ph.D. in Innovation Sciences and Technologies, University of Cagliari, Cagliari, Italy; 2 Department of Medical Sciences and Public Health, University of Cagliari, Cagliari, Italy; 3 Department of Nursing, Popular University of Cesar, Valledupar, Colombia; 4 Department of Mental Health and Addiction, Azienda Sanitaria Locale Mediocampidano-ARES Sardegna, Sanluri, Italy; 5 Department of Education, Psychology and Philosophy, University of Cagliari, Cagliari, Italy; 6 Azienda Ospedaliero-Universitaria Cagliari Servizio di Prevenzione e Protezione Italy, Cagliari, Italy; 7 Instituto de Psiquiatria, Universidade Federal do Rio de Janeiro, Rio de Janeiro, Brazil; 8 Department of Neuroscience, University of Turin, Turin, Italy

**Keywords:** Questionnaire, Confirmatory factor analysis, Human rights, Organizational well-being, Quality of care, Mental health, Caregivers, Psychosocial disability

## Abstract

**Background:**

This study has investigated perceptions of respect for users' rights among informal caregivers in mental healthcare settings, aligning with the guidelines outlined in the United Nations Convention on the Rights of Persons with Disabilities (CRPD) and the World Health Organization QualityRights initiative. The study has employed the questionnaire on Well-being at Work and Respect for Human Rights (WWRR) among informal caregivers and tested whether the questionnaire's factor structure among informal caregivers aligns with that of users and health workers. We have hypothesized that informal caregivers prioritize users' needs and rights over the care context's climate.

**Methods:**

This was a cross-sectional study. The “Well-being at Work and Respect for Human Rights” questionnaire was distributed to 100 caregivers in 4 territorial mental health facilities in Sardinia, Italy. Confirmatory Factor Analysis (CFA) was utilized to assess the participants' responses.

**Results:**

Participants reported high satisfaction with their relatives' treatment, perceiving a high level of respect for human rights among users and healthcare professionals. However, they highlighted insufficient resources for services, particularly the need for additional staff. CFA revealed that a scale with the first five items demonstrated good reliability, convergent validity, and discrimination. Mean scores indicated high satisfaction and perception of respect for human rights across the sample, with no significant differences by age or gender.

**Conclusion:**

Satisfaction with users' rights is closely correlated with other factors comprising the notion of organizational well-being within a healthcare service.

## 1 INTRODUCTION

The United Nations Convention on the Rights of Persons with Disabilities (UN CRPD) underscores the significance of upholding the rights of individuals with disabilities, particularly among people providing care [1]. The UN CRPD is a fundamental step toward changing the negative perception of disability and overcoming the restrictive measures present in the field of mental health and psychosocial disability [2-4]. It requires a shift in how people with psychosocial, intellectual, or cognitive disabilities are viewed by society and in the working methods of mental health and social services. The purpose of CRPD is to ensure that people with disabilities enjoy all human rights and fundamental freedoms, and promote respect for their inherent dignity, individual autonomy, including the freedom to make their own choices, and independence [5]. This underscores the necessity for extensive research on mental healthcare to align with the principles and goals of the UN CRPD, highlighted by the fact that there is also a gap between human rights aims and patients’ experiences in a high-income country [6, 7]. The concept of the UN CRPD has gained prominence in the realm of psychosocial disabilities and garnered global attention through the WHO QualityRights initiative [8-11]. The QualityRights initiative of the World Health Organization (WHO) has been deliberated and approved for use and implementation by government entities in many countries [12-24].

It is crucial for mental health workers to adopt a work approach that respects human rights and trains its practitioners in combating stigma. The research group of the University of Cagliari, which has collaborated with the WHO for the QualityRights project in the Mediterranean area, prioritizes this action as the forefront of their collaboration. However, Saraceno, an authoritative observer, reproached the “avant-garde of rights” due to what happens in the healthcare context. He stated that it is important to start with the implementation of coherent interventions in healthcare settings, rather than simply making predictions [25]. Therefore, consistent with this approach, we have tried, in parallel to the international action, to organize a mental healthcare service attentive to the needs of users, oriented towards recovery, and inclusive of special assistance services. We have also analyzed how aspects of dysregulation in biological and social rhythms, such as sleep and diet, are associated with the onset and course of mental health conditions [26-28], increasing greater awareness of well-being in these areas, which is fundamental to creating capacity, breaking down stigma, and promoting the well-being of the relevant individuals. Perceptions regarding the respect of users' rights, caregivers, and mental health workers are crucial for organizational well-being within mental healthcare settings [29-32].

Indeed, there is a reciprocal relationship between the quality of care in mental health services and the respect for the human rights of users. Violations of human rights can detrimentally affect mental healthcare quality. Conversely, a high level of respect for human rights can enhance the quality of mental healthcare [33, 34]. This notion has formed the basis for the development of the questionnaire on “Well-being at Work and Respect for Human Rights” (WWRR) [35], designed to assess satisfaction with work among mental health workers and care received among users or caregivers, concerning the perception of human rights’ respect within mental healthcare facilities [35]. The WWRR questionnaire aims to raise awareness and introduce the human rights concept due to its simplicity and ease of application. While developed in conjunction with more comprehensive instruments, it is intended as an introductory step to more detailed tools [9, 36-38].

Factor analysis conducted among mental health workers in the Mediterranean and Latin American countries has confirmed the underlying construct, showing a strong correlation between job satisfaction, organi- zational climate satisfaction, and perception of respect for human rights of both health workers and users [39, 40]. Subsequently, the questionnaire was utilized to compare levels of job/organizational satisfaction and perception of respect for rights in the Mediterranean and Latin American regions [41, 42], as well as in Italy in order to compare perspectives on human rights’ respect between users and mental health workers [35, 43], and the satisfaction of mental health users and workers relative to non-mental health service counterparts [44, 45].

Although initially intended for use among non-professional health caregivers, such as family members and volunteers, the questionnaire has yet to be explored within this demographic. This study aimed to address this gap by examining whether the questionnaire's factor structure among informal caregivers aligns with that of users and health workers. We hypothesized that informal caregivers prioritize users' needs and rights over the care context's climate, to which they are somewhat external.

### 1.1 Aim

This study was conducted to establish the reliability and factor structure of the caregivers’ version of the questionnaire on “Well-being at Work and Respect for human Rights” (WWRR).

## 2 MATERIALS AND METHODS

The study was designed according to the Helsinki Declaration of 1975 and subsequent revisions and amendments. The study was endorsed by the institutional review board of the independent ethics committee of the Azienda Mista Ospedaliero Universitaria di Cagliari (Italy) (protocol no. PG/2018/8822 and subsequent amendments).

### 2.1 Design

This was a cross-sectional study. The caregivers of a random sample of patients attending 4 territorial mental health facilities in Southern Sardinia Sanluri, Carbonia, and Cagliari at ASARP and AOU-CA (the same as those recruited for the previous studies on patients and health workers [35, 42]) were asked to complete a booklet containing general socio-demographic information and the “Well-Being at Work and Respect for human Rights” (WWRR) questionnaire. The data were collected between December 2023 and January 2024.

### 2.2 Sample

An informed consent was obtained from all participants in written form. Due to privacy, data concerning individuals who declined participation in the survey were not documented given their failure to return the signed informed consent forms. The sample size of the study was determined based on previous studies involving service users and health workers with similar methodologies and measurement tools. This was done to ensure that the study had sufficient power to detect significant effects or associations between the variables of interest. The ultimate sample comprised 100 caregivers (Table **1**).

### 2.3 Measures

The booklet included a section on socio-demographic data, including age, gender, education, and civil and occupational status. Then, there was the WWRR questionnaire.

#### 2.3.1 “Well-being at Work and Respect for human Rights” (WWRR) Questionnaire

The questionnaire is a component of the broader global initiative led by the World Health Organization, which emphasizes human rights and the successful enforcement of the UN CRPD, referred to as the QualityRights initiative (https://www.who.int/mental_ health/policy/quality_rights/en/) [46-49]. Its primary objectives include measuring how patients and staff perceive respect for human rights within healthcare settings and its association with organizational and working climates. Developed through collaborative efforts with professionals, such as psychiatrists, psychologists, rehabilitation technicians, and psychometrists, the scale aims to provide a concise and user-friendly assessment tool suitable for potential utilization in extensive multicenter research endeavors. The WWRR is expected to measure a single latent trait of satisfaction with well-being, with respect to human rights being closely correlated to it [39]. The WWRR has undergone translation from Italian into English, French, Macedonian, and Maghreb Arabic. The questionnaire primarily comprises six core items, with a seventh item serving an exploratory purpose, as it may offer insights into the perceived resource needs of different personnel or teams (“Which types of professionals do you think would be most useful to add to the service where your relative is cared for?: doctors, psychologists, nurses, educators or rehabilitation technicians, social assistants, support staff, security personnel”) [39]. It is tailored for implementation by healthcare practitioners across a broad spectrum of healthcare settings.

The formulation of the questions for caregivers is reported in Table **2**.

### 2.4 Data Analysis

The data were analyzed with the Statistical Package for the Social Sciences (SPSS) version 29 (IBM Corp. Released 2021) and packages running in R [50]. All analyses were two-tailed, with significance set at p <0.05.

Categorical variables were depicted as counts and percentages, whereas continuous variables were characterized by means along with their respective standard deviations and ranges.

We applied Confirmatory Factor Analysis (CFA) to the caregivers' version of the WWRR to evaluate its unidimensionality. Mardia’s test [51] showed a deviation from multivariate normality in the data (skewness=169.4; p<0.0001; small sample skewness=176.3; p<0.0001; kurtosis=4.1; p<0.0001), and the scores were ordinal. Thus, the Weighted Least Square Means and Variance adjusted (WLSMV) estimator was used in CFA.

The following parameters were used to assess goodness-of-fit: the chi-square, the Comparative Fit Index (CFI), the Root Mean Square Error of Approximation (RMSEA), and the Standardized Root Mean square Residual (SRMR). When chi-square indicates a deviation from goodness-of-fit (p<0.01), still an acceptable fit can be stated with RMSEA values of 0.08 or lower, SRMR values of 0.09 or lower, and CFI values of 0.90 or higher [52].

The following indicators of reliability were estimated from the model: Cronbach’s alpha and McDonald's omega [53]. Items were expected to load onto the unidimensional factor with at least 10% of explained variance (≥.32 loading).

Optimal discrimination was deemed for item-total correlation with values >0.40. Finally, the Average Variance Extracted (AVE) was calculated to determine how much variation in the items was explained by the latent variable, with AVE>0.50 being the minimum threshold for convergent validity [54].

## 3 RESULTS

The final sample included 100 caregivers, including about two-thirds of women. The mean age was 55±13, ranging from 18 to 82 years old. Men were on average older than women: 60±10 *versus* 52±13 (Student t-test; p=0.006). About 60% of participants had a high school or university degree. Most participants were married (63%), while 15% were either separated, divorced, or widowed. Only 42% of the sample were fully employed, while 19% were housewives and 21% were retired (Table **1**).

A new model was tested by exclusion of item 6. Goodness-of-fit remained acceptable based on the parameters, and an important improvement of the reliability was found, with McDonald's omega approaching the best threshold (Table **3**).

The loading of the items did not change, with item 5 showing the lowest loading on the unidimensional factor, but AVE showed an increase, reaching the threshold for acceptance (Table **4**).

A scale with the first five items was likely to show good convergent validity and discrimination based on item-total correlation and AVE.

### 3.1 Mean Scores in the Sample

The scores for the five-item WWRR were computed by summing the responses to each item and dividing by the total number of items, resulting in a scale ranging from 1 to 6. Higher scores on the questionnaire reflect increased satisfaction or perception of respect for human rights. Mean scores in the sample were 5.2±0.8, ranging from 2.2 to 6. Just 6% of the sample scored less than 4 on the five-item WWRR.

No link with age was found in the sample (Spearman's rho=0.06, p=0.56). Men (5.0±0.8) and women (5.2±0.7) were equally satisfied with the service in which their relatives were cared for (t-test=-1.37, p=0.17).

## 4 DISCUSSION

The study has confirmed WWRR to measure a single latent trait primarily linked to satisfaction with the service and the perception of respect for human rights. The factorial structure of the WWRR among caregivers has been found to be in line with previous adaptations. The construct has been found to be a single latent trait correlating with all items. Conceptually, the main axes of the hypothesis have been confirmed, namely, organi- zational well-being correlating with the perception of respect for users’ rights; however, caregivers have perceived item 6, related to service resources, to be less correlated. As anticipated, caregivers have prioritized users' needs above the climate of care contexts. This has led to the optimal fit for a five-item model of the WWRR, demonstrating satisfactory reliability and good convergent validity and discrimination.

Overall, participants have shown to be reasonably satisfied with the service in which their relatives are cared for, with no relevant differences by age or gender.

As in past studies concerning mental health workers [39, 40], a substantial link was observed between satisfaction with the work organization and perception of respect for human rights within the work organization, it was, in this case, measured as satisfaction for the care received by the caregivers' relative.

However, unlike the papers on the factorial structure of the questionnaire applied to mental health workers [39, 40], item 6 did not appear to define a single component, with respect to the previous items. The link with item 5 was also substantially low.

In essence, for workers and users, the scarcity of resources is a factor related to organizational satisfaction and respect for user rights [35, 42]. However, for the service staff and informal caregivers, the perception of respect for workers' rights also maintains a loose link with organizational satisfaction and respect for users' rights.

This element can be explained precisely in consideration of the particular perspective of the caregivers. They are more centered on the well-being of their relative/friend/care recipient and are probably not as inclined to see respect for the rights of health workers as very important for this purpose. Even with respect to the problem of resource scarcity, it is more likely that those on the front line (users and workers) can have a clearer vision of how this element is relevant to the general and organizational well-being of staff and respect for rights.

The caregiver, often a relative of the user, is therefore more sensitive to the lack of resources within a service while still perceiving a good climate and quality of services. This phenomenon could also be explained by the unique Italian context, in which mental healthcare is predominantly delivered in community settings and within society. Nonetheless, the caregiver still significantly reports the lack of resources, indicating that an imminent problem could arise concerning the quality of care and organizational well-being of the service due to resource scarcity. Despite this discrepancy, the main axes of the hypothesis, namely organizational well-being and the perception of respect for users’ rights, maintain a significant correlation, confirming the general concept.

This study has involved the obvious limitations of having been conducted in a voluntary sample coming from a single large geographical area. In addition, the WWRR addresses broad issues that deserve to be detailed and explored in depth. This tool is designed to raise awareness and introduce human rights concepts as it is easy and quick to apply. It has been developed alongside other, more in-depth instruments, but it is intended to serve as a preliminary step to these more comprehensive tools [9, 36-38].

## CONCLUSION

In conclusion, satisfaction with users' rights is closely correlated with other factors comprising the notion of organizational well-being within a healthcare service.

Future studies need to measure in a more widespread and generalizable way the caregivers' point of view on how respect for the human rights of users and mental health workers can influence organizational well-being and satisfaction with care in mental health services.

It is also important to compare the points of view of caregivers, users, and mental health professionals on these issues. Any discrepancies must be the subject of in-depth analysis and discussion as an essential moment in the process of continuous improvement of care.

## AUTHORS’ CONTRIBUTION

All authors have accepted responsibility for the manuscript's content and consented to its submission. All of them have meticulously reviewed the results and unanimously approved the final version of the manuscript.

## Figures and Tables

**Table 1:** Characteristics of the sample.

-	-	n = 100	
**Gender**	Men Women Undeclared	37 (37%) 62 (62%) 1 (1%)	
**Age**	Mean (SD); range	55 (13); 18 to 82 years old	
**Education**	Elementary school Middle school High school University degree Master/PhD/specialization Undeclared	9 (9%) 31 (31%) 42 (42%) 13 (13%) 3 (3%) 2 (2%)	
**Civil status**	Single Married Separated/divorced Widower/widow Undeclared	20 (20%) 63 (63%) 9 (9%) 6 (6%) 2 (2%)	
**Occupation**	Unemployed Student Housewife Employed Retired Disabled Undeclared	8 (8%) 3 (3%) 19 (19%) 42 (42%) 21 (21%) 1 (1%) 6 (6%)	

**Table 2:** Distribution of the scores by item of the caregivers’ version of the “Well-Being at Work and Respect for human Rights” (WWRR) questionnaire.

Item	Mean (SD); Range	% Scoring Low*	Item-total Correlation
1. “How satisfied are you with the care your relative receives?”	5.2 (0.9); 2 – 6	2%	0.67
2. “How satisfied do you think that the patients of the service where your relative is treated are with the treatment they receive?”	5.1 (1.0); 2 – 6	4%	0.64
3. “How satisfied are you with the organization of the service in which your relative is supported?”	5.1 (1.2); 1 – 6	1%	0.57
4. “How much do you think the human rights of patients are respected in the care service where your relative is cared for?”	5.2 (1.0); 1 – 6	1%	0.60
5. “How much do you think are the human rights of the workers respected in the service where your relative is cared for?”	5.3 (0.8); 2 – 6	1%	0.35
6. “How do you evaluate the treatment situation in the service where your relative is cared for, with reference to the available resources?”	3.6 (1.5); 1 – 6	29%	0.02

**Note: **Expected scores for question 1 to 3: Likert scale, from 1 (“not at all”) to 6 (“completely satisfied”).

Expected scores for question 4 and 5: Likert scale, from 1 (“not at all”) to 6 (“completely respected”).

Expected scores for question 6: Likert scale from 1 (“The resources are adequate”) to 6 (“Serious resource deficits”) [9].

* A low scoring is assigned for replies 1 or 2 on questions 1 to 5, and for replies 5 or 6 on question 6.

**Table 3:** Confirmatory factor analysis of the body shape questionnaire (BSQ). Goodness-of-fit indices of the tested models using the weighted least square means and variance adjusted (WLSMV) estimator.

Model	χ^2^	df	p	CFI	RMSEA (90%CI)	SRMR	Cronbach’s α	McDonald’s ω
6-item	18.54	9	0.029	0.959	0.068 (0.021 – 0.112)	0.071	0.691	0.728
5-item	16.26	5	0.006	0.959	0.094 (0.045 – 0.146)	0.079	0.830	0.847
-	-	-	-	-	-	-	-	-
Threshold for fit	-	-	p>0.05	>0.90	<0.08	<0.09	≥0.70	≥0.90

**Table 4:** Factor loading of the items of the caregivers’ version of the “well-being at work and respect for human rights” (WWRR) questionnaire [9] in the 6-item and the 5-item model.

Item	6-item Model	5-item Model
1. “How satisfied are you with the care your relative receives?”	0.82	0.82
2. “How satisfied do you think that the patients of the service where your relative is treated are with the treatment they receive?”	0.81	0.81
3. “How satisfied are you with the organization of the service in which your relative is supported?”	0.76	0.76
4. “How much do you think the human rights of patients are respected in the care service where your relative is cared for?”	0.75	0.75
5. “How much do you think are the human rights of the workers respected in the service where your relative is cared for?”	0.35	0.35
6. “How do you evaluate the treatment situation in the service where your relative is cared for, with reference to the available resources?”	0.02	---
AVE	36.6%	55.0%

## Data Availability

The authors confirm that the data supporting the findings of this study are available within the article.

## LIST OF ABBREVIATIONS

CRPD: Convention on the Rights of Persons with Disabilities
WWRR: Well-being at Work and Respect for Human Rights
CFA: Confirmatory Factor Analysis

## STANDARDS OF REPORTING

STROBE guidelines were followed.
